# “I will not accept to bury my pig”: *Taenia solium,* WASH, and gender roles in the smallholder pork value chain in Southern Province, Rwanda

**DOI:** 10.1371/journal.pntd.0014815

**Published:** 2026-10-21

**Authors:** Janna M. Schurer, Ruth Zihiga Uwase, Prince Rwagasore, Kristen Rodrigues, Anselme Shyaka, Tayechalem Moges

**Affiliations:** 1 Center for One Health, University of Global Health Equity, Butaro, Rwanda; 2 Veterinarians without Borders Canada, Ottawa, Ontario, Canada; 3 Center for Gender Equity, University of Global Health Equity, Butaro, Rwanda; Zhejiang Wanli University, CHINA

## Abstract

*Taenia solium* is a zoonotic cestode and the causative agent of human taeniasis/cysticercosis and porcine cysticercosis. The burden in Rwanda is significant, including death and disability in people and income losses across the pork sector. Despite widespread recognition on the need for multi-sectoral and inclusive *T. solium* control programming, little is known about water, sanitation and hygiene (WASH) access and gendered disease exposure among rural smallholders involved in the formal and informal pork sectors. This study aimed to characterize gender roles and WASH access in the Nyamagabe District (Southern Province) pork value chain (PVC) to better inform future interventions for disrupting *T. solium* transmission. We conducted Focus Group Discussions with 23 men and 21 women across five nodes of the PVC (farmers, animal health workers, butchers, vendors, consumers) and interviewed six key informants. Audio-recordings were transcribed verbatim, translated into English and coded as part of inductive thematic analysis. PVC participants identified numerous biosecurity gaps in the Nyamagabe PVC, such as free-range pig production, re-use of human waste, lack of prevention services (deworming, vaccination), informal slaughter, and consumption of infected and/or undercooked pork. Traditional gender roles were prevalent, with women managing domestic roles related to pig husbandry and men managing roles related to building pens, buying/selling pigs and slaughtering/butchering. Water and sanitation facilities were frequently inadequate, causing serious lapses in human and pig hygiene. Participants identified poverty as a major barrier for implementing *T. solium* control but demonstrated enthusiasm for receiving information and resources to support this goal. Altogether, these findings support the need for One Health programming to reduce the burden of *T. solium* and other zoonotic diseases in people and pigs in Rwanda. Gendered norms among rural smallholders indicated a need for gender transformative information campaigns targeting specific recipients along the PVC nodes.

## Author summary

*Taenia solium* is a parasitic tapeworm that infects both people and pigs. This parasite is a serious concern due to human morbidity and mortality among infected people, and income losses to pig producers. Burden reduction requires a One Health approach, improving water and sanitation access, deworming people and pigs, educating stakeholders, and improving biosecurity measures. However, rural smallholder pig farmers often lack such services. Our study aimed to understand how men and women’s roles differed along five nodes of a rural pork value chain in Southern Rwanda and to characterize their access to water and sanitation. We hosted Focus Group Discussions with male and female farmers, animal health workers, butchers, vendors, consumers (n = 44) as well as various key informants (n = 6). We learned that women primarily cared for pigs while men built pens, slaughtered pigs and controlled finances. Key biosecurity gaps among rural smallholders included allowing pigs to eat human waste, evading veterinary inspection during slaughter, lack of pig deworming/vaccination services, and consuming undercooked pork from infected pigs. Poverty and poor access to water and sanitation impeded efforts to control *T. solium.* Altogether, this indicates the need for gendered community education and multidisciplinary veterinary public health solutions for pork value chain actors.

## Introduction

*Taenia solium,* aka the pig tapeworm, is a leading cause of foodborne illness, responsible for up to one-third of adult-onset epilepsy in sub-Saharan Africa (SSA) [1–3]. In the natural lifecycle, humans carry the adult cestode in their small intestines, shedding infective proglottids through fecal matter, which is later eaten by pigs. These pigs develop metacestodes (cysticerci) in their muscle, organ, and neural tissues which re-infect people through the consumption of raw or undercooked pork [4]. Humans who accidentally ingest *T. solium* eggs are at risk of neurocysticercosis (NCC), a serious and debilitating neurological condition that occurs when metacestode stages develop in the central nervous system [1]. One Health approaches, considering human, pig, and WASH (water, sanitation and hygiene) factors for transmission, interrupt the *T. solium* lifecycle at multiple points, offering stronger chances for successful mitigation.

In Rwanda, the *T. solium* burden is not well characterized, relying mostly on point estimates. Porcine cysticercosis appears to be concentrated in the south-west, with 25–43% of pigs infected, compared with 2–3% elsewhere in the country [5]. This estimate was based on sero-positivity of a commercial antigen-ELISA (Enzyme-linked Immunosorbent Assay) and validated by whole carcass dissection in a sample subset. Among children, taeniasis prevalence ranged from 0.3% in the north, as diagnosed through Kato-Katz stool analysis and confirmed by coproantigen ELISA, to 1.4% in the west using an unspecified stool analysis [6,7]. A sero-positivity of 13.3% was reporting in the same Northern Province children using enzyme-linked immunoelectrotransfer blot [7]. As elsewhere, NCC is strongly correlated with epilepsy [8]. Risk factors broadly associated with *T. solium* infection include living in endemic regions, co-habitation with free-roaming pigs, low education, poor access to improved WASH, and ingesting raw or under-cooked pork [1,8]. The relative risk of infection between men and women is unclear, but well-documented differences in rural men’s and women’s household responsibilities suggest differing transmission pathways [9–11]. Economic losses attributed to *T. solium* are predominantly due to NCC related productivity losses, illness and death in people, as well as pig production losses [12]. In southern Rwanda, traders inspect pig tongues by manual palpation, lowering the purchase price by 5000 RWF for each cyst found [13].

Reducing the burden of *T. solium* requires multi-sectoral programming that integrates WASH and other environmental management strategies with veterinary public health initiatives [1,14]. Improved sanitation facilities are a critical tool for disrupting the *T. solium* lifecycle, sequestering human excreta from contact with both people and pigs. However, unimproved household sanitation, public defecation, and informal slaughter under low biosecurity continue to occur in rural areas of Rwanda with high pig production [13,15,16]. Moreover, small-scale producers are more likely to allow free grazing of pigs [13], raising the risk that pigs will ingest human stool and develop cysticercosis infection. This practice is prohibited under Gazette n°50 of 13/12/2010 but remains widespread. Clean water and access to soap also reduce the risk of human cysticercosis and NCC through safer food preparation and good hand hygiene practices. Across Rwanda, 82.3% of households have access to improved drinking water, but this drops to 55.9% in rural areas of Southern Province where *T. solium* is endemic [16]. Handwashing with soap in such areas is inconsistent, primarily due to financial difficulties of buying soap and poor knowledge of disease prevention [15].

The Government of Rwanda has set clear goals to reduce *T. solium* related morbidity and mortality by 25% [17]; while at the same time, announcing plans to increase pork production by 239% [18]. In the absence of strict programming to reduce *T. solium* transmission, such as human deworming, pig vaccination/treatment and improved sanitation, there is a serious likelihood that such rapid increases in pig production will negatively impact human and animal health, in addition to compromising country goals for poverty reduction and enhanced international trade [14,19]. Therefore, our study team initiated a 4-year program (Community One Health Empowerment in Rwanda and Senegal - COHERS) to explore cost-effective interventions for reducing the burden of *T. solium* cysticercosis in Rwanda. This baseline study aimed to explore gender roles and WASH practices along the pork value chain in the poorest district of Rwanda, as well as to characterize *T. solium* knowledge and information sharing between production nodes. Collecting such gender disaggregated data on WASH access, participation in pork production, and differences in transmission risk is a necessary prerequisite to developing sustainable strategies for reducing cysticercosis burden at a time of rapid agricultural expansion.

## Methods

### Ethical statement

Ethical approval for this study was provided by Rwanda National Ethics Committee (51/2024). Prior to data collection, the study team obtained permission to work in Tare Sector from district and sector officials. All participants provided written informed consent before engaging in this study.

### Study setting

Nyamagabe District, located in Southern Rwanda, is home to more than 370,000 people, of which 52.7% are women [16]. It has the highest poverty rate in Rwanda (51.4%) and the second highest rate of food insecure households (42%) [20,21]. Most households engage in crop cultivation (81.9%) and/or livestock production (72.2%), with cattle and pigs being most common [16]. Nyamagabe is a hilly region that suffers frequent floods and landslides. Among rural households, 44.1% are reliant on unimproved water sources and most use pit latrines that are either family specific or shared between households. Unimproved WASH infrastructures are those that do not adequately protect people and animals from contact with raw waste, due to contaminated water or leakage from sewage systems [22]. Nearly one-third of households are female-headed (30.7%) [16]. We chose to conduct this study in Tare Sector (Nyamagabe District) due to the high endemicity of porcine cysticercosis, the popularity of pork production, and the presence of the largest pig market in Southern Province.

### Study design and participant recruitment

This descriptive qualitative study employed focus group discussions (FGDs) and key informant interviews (KIIs) to characterize gender inclusion and WASH access along five nodes of pork production: namely, pig producers, venders (those who sold live pigs or cooked pork), butchers, community animal health workers (CAHWs), and consumers. This qualitative approach complemented a large-scale quantitative assessment of household *T. solium* prevalence and WASH access, intending to document structural and behavioural drivers of ongoing disease transmission with greater depth, allowing flexibility to explore unexpected findings in an under-researched region. All data was collected in May 2025. We conducted one FGD for each of the five value chain nodes, recruiting 6–10 men and women per FGD. Mixed gender groups were considered appropriate as past experiences and pre-testing demonstrated that both groups were willing to engage freely in pork value chain discussions. Key informant interviews were conducted, as needed, to triangulate information provided in FGDs. Study participants were purposively selected by first identifying key individuals through discussions with Tare Sector community leaders and then using snowball sampling from FGD participants to identify other relevant stakeholders. Community leaders were requested to identify diverse groups of individuals representing differences in PVC participation, age, gender and geographic location. Those eligible were adult residents of Nyamagabe District who had worked in a specific node of the pork value chain for at least one year. FGD facilitators (R.Z. and P.R.) debriefed after each interview to identify ongoing data gaps and discuss emerging themes. Data collection continued until the entire team agreed that thematic saturation had been reached.

### Data collection

Semi-structured FGD and KII guides were developed in English by a team of local and international scientists, with expertise in veterinary medicine, parasitology, pork production, gender, and WASH. Major themes included: (1) gender inclusion in the pork value chain, (2) WASH access, (3) *Taenia solium* knowledge, (4) willingness to pay, and (5) information sharing. It was later translated into Kinyarwanda and pre-tested with pig farmers and health workers outside the target region in Butaro District. FGDs lasted approximately 100 minutes and were conducted by R.Z. and P.R. in Kinyarwanda. All sessions were audio-recorded.

### Data analysis

FGD and KII audio recordings were transcribed verbatim and translated from Kinyarwanda to English by trained enumerators. Thematic analysis included open reading of all transcripts by two researchers (T.M and R.Z.) to develop a codebook, followed by inductive coding in Dedoose (Version 9.2.14) using the constant comparison method. Consensus validation was used to refine the codebook and finalize coding through multiple rounds of discussion. In the case of disagreement, a third team member could be consulted to mediate an agreement. Finally, representative quotations were used to illustrate study findings. The study methodology and results were developed according to the Standards for Reporting Qualitative Research (SRQR) checklist [23].

## Results

Five FGDs were conducted with 23 men and 21 women (N = 44), including 10 pig producers, 6 pork vendors, 9 pork butchers, 9 CAHWs, and 10 pork consumers (Table 1). Six KIIs were held with sector veterinarians (n = 3), a registered nurse (n = 1), a slaughterhouse owner (n = 1), and a district-level hygiene and sanitation officer (n = 1). Only one key informant was female. Participant age ranged from 24 to 61 years with a mean of 41 years.

**Table 1:** Focus group and key informant pork value chain participants (N = 50).

Participants	Males n (%)^1^	Females n (%)	Total n (%)
*Focus group discussions*			
Pig producers	3 (6)	7 (14)	10 (20)
Pork vendors	5 (10)	1 (2)	6 (12)
Butchers	6 (12)	3 (6)	9 (18)
^2^CAHWs	3 (6)	6 (12)	9 (18)
Pork consumers	6 (12)	4 (8)	10 (20)
*Key informants*			
Sector veterinarian	3 (6)	0 (0)	3 (6)
Registered nurse	0 (0)	1 (2)	1 (2)
Slaughterhouse owner	1 (2)	0 (0)	1 (2)
^3^District WASH officer	1 (2)	0 (0)	1 (2)
**Total**	**28 (56)**	**22 (44)**	**50 (100)**

^1^Calculated as percent of total sample; ^2^Community animal health workers; ^3^Water, sanitation and hygiene.

Analysis of FGD and KII transcripts identified three major themes:

1. Gendered social norms along the PVC contribute to differential disease risk
2. Inadequate WASH access in public and private facilities facilitate *T. solium* transmission
3. Education gaps impact community disease control efforts

### Theme 1: Gendered social norms along the PVC contribute to gendered disease risk

#### Production.

Both men and women were involved in raising pigs. Men constructed pigsties, purchased inputs such as animal feeds and medications, and made all major financial decisions. Women fed pigs, cleaned pens, treated sick pigs, and arranged pigsty construction through hired labour. Children helped with household chores, with boys responsible for taking pigs outside to graze. A few young men took on roles traditionally reserved for women, such as cleaning pens and feeding pigs but these shared responsibilities were considered unfeasible for other men who left their homes to earn income.

*“When the man has gone to look for work, …the woman must take care of the domestic animals.*” FGD, CAHWs, Female participant (FP)

*“Previously, piggery was for women. Men never touched pigs. But now you find even men cleaning and feeding them.*” FGD, Farmers, Male participant (MP)

#### Animal health.

Animal health personnel were perceived as being too scarce to meet the needs of the population. Male animal health workers were considered to be stronger, braver, able to travel long distances, more available and the preferred (traditional) choice, especially when treating cattle. Women were not available in the morning due to domestic responsibilities and were generally trusted only to care for “women’s animals” (small ruminants, poultry). In one case, a female veterinarian challenged this stereotype by checking on a sick cow each morning, demonstrating both availability and capacity to provide quality care. Women acknowledged the double burden of unpaid domestic labour and caring for household pigs, noting that their inclusion into animal health professions was increasing. Veterinarians enjoyed a high level of trust from livestock producers but were not affordable for smallholders. They were generally only called to large farms or seen during mass vaccination events organized by the government. CAHWs could not charge for their services and were often the first point of contact when a farmer had a sick pig. Men and women appeared to have equal access to veterinary services.

*“In most cases, 95% are men…. They are the ones who take on the veterinary profession.”* KII, Veterinarian, MP

*“Sometimes you find it’s the female veterinary who is nearby and therefore it is necessary that they call her to support… when there is another one who is male, they prefer the man to the woman.”* KII, Veterinarian, MP

*“I called a self-employed female veterinarian…She made it her responsibility to check on the cow every morning.”* FGD, CAHWs, FP

#### Slaughter.

Butchering was considered an exclusively male domain. Both men and women agreed that the tools to slaughter animals and butcher meat were dangerous for women. Moreover, they felt that women’s caregiving duties and compassionate nature made them unsuitable for such work. Men slaughtered pigs, butchered meat and sold pork while boys assisted by cleaning slaughter areas and tools. Pigs were usually slaughtered at the point of sale (i.e., restaurants rather than abattoirs). Those women who sold raw or cooked pork hired men to butcher the pigs.

*“When it comes to slaughtering, women are compassionate and fearful… No woman would be happy to slaughter an animal. However, women who run pork restaurants often have others butcher pigs for them.”* FGD, CAHWs, FP

*“The boys we work with clean the slaughter slab and tools.”* FGD, Butchers, MP

#### Vendors.

Women sold pigs in their own villages but did not often travel to markets where men dominated pig trading as professional vendors (Fig 1). Men and women stressed that household decisions, such as whether to sell a pig, were often discussed but that the final decision lay with the man. Men considered women to be poor negotiators, accepting lower prices in part because they were less well informed about pig diseases. They felt women were not strong enough to walk or carry pigs long distances to the markets and should stay home to focus on domestic responsibilities. Women were considered more financially responsible, using pig earnings on family expenses (e.g., school fees), while men felt that market days should include social activities such as drinking beer.

**Fig 1:**
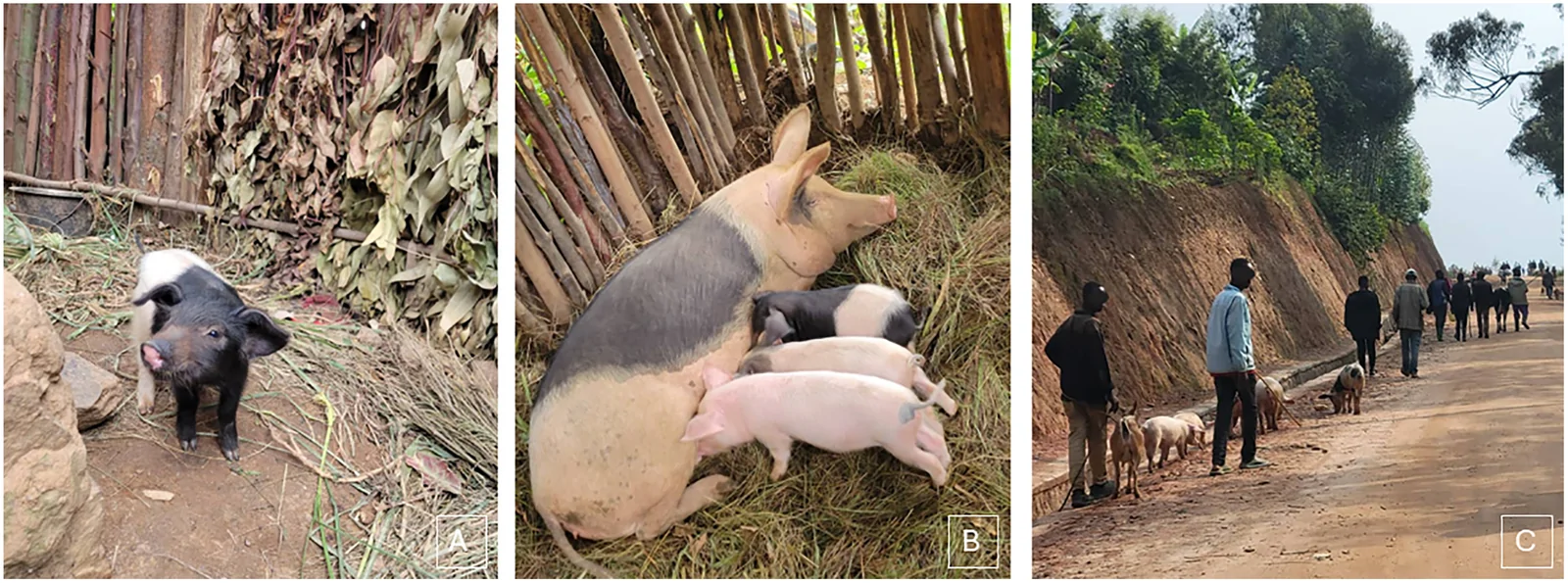
Scenes from the pork value chain in Nyamagabe District, Southern Province, Rwanda. A: Free-roaming piglet, B: Confined sow and piglets, C: Men bringing pigs to market (Photo credit: J.M. Schurer).

*“The majority of vendors are men because women cannot lift it when it falls in water…women also do not know how to check for pig cysticercosis.”* FGD, Butchers, MP

*“When I go somewhere else to work a temporary job and earn one or two thousand francs, I should take a thousand for home expenses and another for drinking beer”* FGD, Consumers, MP

#### Consumption.

Pork was consumed by both men and women; however, participants disagreed on whether men consumed pork meat in public places more often than women, indicating a change in women’s consumer practices. Participants agreed that pork was expensive and that most farmers were too physically tired after a long day to eat out frequently. Women consumed *ibikoco* (pork skin) more often than pork meat (due to affordability) and were seen as better cooks. Children only consumed pork when it was brought home.

*“In the centers, to find a woman who has gone to eat meat is very uncommon…they don’t get time to go to pork places.”* KII, Veterinarian, MP

*“Women are consumers of pork…I receive 50% of women consumers and 50% of men consumers.”* KII, slaughterhouse owner, MP

### Theme 2: Inadequate WASH access in public and private facilities facilitate *T. solium* transmission

#### Subtheme 1: Safe water and sanitation infrastructure.

Participants reported inadequate water and sanitation infrastructure across all nodes of the pork value chain, but especially in rural areas. Even when water infrastructure existed, access could be difficult due to damaged pipes, long distances, long waiting times, or unaffordable services. This drove some households and vendors to obtain and use untreated water. It meant that women were also frugal, reserving leftover dishwater for their pigs.

*“Some places have water, others don’t. Old pipelines are damaged and hard to repair.”* KII, Veterinarian, MP

*“If someone has to spend two hours to get drinking water, they will take whatever they can get without considering its source.”* FGD, Consumers, MP

In private homes, latrines could be poorly constructed, lacking doors or completely absent. Few public slaughterhouses had well-constructed latrines that were clean and in good working order.

*“There are no toilets here. In reality, you find households without toilets, … even rented houses have none.”* KII Slaughterhouse owner, MP

*“Most homes don’t have standard sanitation. Flies come from poor latrines and land on the meat.”* KII, Veterinarian, MP

*“We have public toilets in only three sectors in good condition. Others are either old or inadequate.”* KII, WASH officer, MP

#### Subtheme 2: Gender and hygiene.

Good hygiene related to pig production, latrine management and food safety practices was considered women’s responsibility. Women fetched water and conducted day to day cleaning with the help of their children, while men were tasked with constructing infrastructure. Both were affected if water was not available. Attitude was considered a key factor in maintaining good hygiene practices.

*“There’s a belief that cleanliness is a woman’s responsibility.”* KII, WASH officer, MP

*“There isn’t much difference [in terms of cleanliness] between men and women… If there’s no water at home, neither men nor women will be able to wash.”* KII, Veterinarian, MP

*“Even those [men] with income spend it on beer while their homes remain filthy, it is about priorities.”* FGD, Consumers, MP

Managing human waste was a challenge both at home and in public. Lapses in appropriate sanitation facilities encouraged individuals to engage in socially unacceptable practices, namely public defecation, using human waste as fertilizer, and feeding human waste to pigs. Men were considered more likely to practice public defecation, possibly due to the longer distances they travelled from home. In some homes, human waste was composted and then applied to food crops as fertilizer.

*“Men often go in the bush; we women try to hide and borrow toilets if we can.”* FGD, CAHWs, FP

*“We cover it …once it decomposes…use it as fertilizers.”* FGD, vendors, MP

Feeding human waste to pigs was reported by several sources, practiced by both men and women, and generally hidden from local leaders. Usually, it occurred accidentally, when people defecated in open areas occupied by free-roaming pigs. When deliberate, waste was mostly fed raw. Those involved identified a win-win benefit – pigs were fed and compounds were cleaned from human waste. This disincentivized people to build proper latrines or to obtain forage for pig feeds. Others felt that the government should intervene to stop such practices. Participants overall felt that this practice was decreasing.

*“I will not waste my feces; I have a pig that should eat.”* FGD, Consumers, MP

*“Women are the ones with small children who might defecate anywhere….so they clean and feed the pigs.”* FGD, Consumers, MP

*“They go there at night, and in the morning, they send their pig there to eat the feces… Some people hide this from local leaders.”* FGD, Consumers, MP

#### Subtheme 3: Biosecurity and food safety adherence.

Participants along the PVC were generally aware of regulations related to pig production, slaughter facilities, veterinary inspection and WASH. For producers, poverty and the need to provide for families was a strong disincentive for adherence. For example, some allowed pigs to share their living spaces, while others purposefully circumvented veterinary inspection of their pigs at slaughter to avoid financial loss. Government veterinarians stated that they were not allowed to confiscate diseased pigs from houses or marketplaces, highlighting this regulatory gap as a key weakness in controlling *T. solium*. Veterinarians were only mandated to inspect pork at slaughterhouses and could only impose fines or condemn carcasses at that point. Consumers agreed that it was common practice to eat the meat from pigs that died suddenly, were diseased, or did not undergo inspection. In some instances, pigs that had been condemned and ordered buried by the veterinarian were exhumed hours later and consumed. When an outbreak of an infectious disease such as African swine fever occurred, producers might sell one pig to finance the vaccination or treatment of others.

*“Pork is not just eaten at restaurants… When the pig dies …they slaughter and share the meat among neighbours.”* FGD, Consumers, FP

*“There is poverty. I will not accept to bury my pig because of an infection. I will do my best to get a small interest”* FGD, CAHWs, FP

*“When a resident finds out their pig is infected, they might say they’ll take it back home, but often, they end up selling it secretly instead. We can’t confiscate the pig because it’s their property, and there’s no legal way to take it from them. If they choose to take it home, we have no authority to stop them, which makes controlling the spread of the disease difficult.”* KII, Veterinarian, MP

*“If there are signs of African swine fever, we can sell a piglet in order to get money for vaccination of the remaining pigs.”* FGD, Butchers, FP

Veterinarians and CAHWs identified good biosafety protocols, such as wearing gloves while examining animals and washing hands between farms, but did not always adhere to them when water or personal protective equipment was in short supply. Many villages had no local access to a properly constructed slaughter slab. This compelled butchers to slaughter animals under unhygienic conditions, where water was inadequate to keep surfaces, instruments and meat clean. Offal was fed to other animals or buried in large pits. Some vendors lied about the origin of pork cuts, putting a healthy carcass on display but selling diseased meat.

*“There are no proper slaughterhouses…many others may use improvised areas in the bushes.”* KII, Veterinarian, MP

*“If there’s no water at the herd place, I look for a nearby location where I can access water. Often, the herd keeper might say there’s no water available on-site. In such cases, I need to find water outside the herd area to wash my hands. However, most of the time, water is not available”* KII, Veterinarian, MP

*“These pigs are then sold alongside those that have been properly inspected and tested by veterinarians. … you might see healthy-looking pigs that have been tested hanging there, but there are also others that have been hidden in rooms and mixed in, posing potential risks to consumers.”* FGD, Consumers, MP

In public eating establishments, inadequate WASH infrastructure was associated with poor food safety practices, such as cutting meat on dirty surfaces, storing meat at room temperature and reusing dirty crockery between customers. Some vendors were noted to wash clothing infrequently.

*“He [a vendor] doesn’t wash clothes, he doesn’t change clothes. He may spend one week without changing.”* FGD, Farmers, MP

*“There are several challenges we face. The first is water shortage. If people don’t have water, they won’t properly wash the plates they use to serve food. When water is scarce, they might use the same plate multiple times without cleaning it. This problem is compounded by the long distances they must travel to fetch water, and the fact that we don’t monitor their kitchen practices… The seller hides the infection, and the meat ends up being consumed by people, which is a significant problem we encounter as veterinarians.”* KII, Veterinarian, MP

Pork was fried, boiled or roasted depending on taste preferences and access to water and oil. Some participants reported not washing raw pork to retain fat. Traditionally, only the pork muscle was consumed but participants identified a change to consume all parts of the pig, highlighting that this might increase disease transmission risk. A common concern was the ingestion of undercooked or raw pork. A recent government campaign advised consumers that deep frying reduced the risk of disease transmission more than roasting and that meat should not be prepared on skewers. Misperceptions were also prevalent. For example, men were perceived as more protected because of their ability to pay for alcohol that protected them from infection after consuming pork.

*“Before they used to slaughter and throw away the offal [intestine organs] but now after slaughtering they are removed washed and called meat.”* FGD, CAHWs, FP

*“People were eating raw pork thinking it was ready when it wasn’t.”* FGD, Vendors, MP

“*Women are more infected because they immediately drink water after consuming pork. But men have low risks of infection because they don’t drink water after consuming pork..., they take alcoholic drink.*” FGD, Farmers, FP

### Theme 3: Education gaps impact community disease control efforts

#### Subtheme 1: Unequal access to information.

Human, animal and environmental health information was obtained from health centers, community meetings, Community Health Workers (CHWs), local media (radio, television), cars with loudspeakers, and non-governmental organizations (NGOs). Radio was a preferred media but more accessible for men. Women were more frequently reached through community meetings and health services. Participants disagreed on who had more time to attend information meetings. Some felt that women were disadvantaged due to domestic responsibilities and their need to ask permission from husbands to attend. Others felt that men were too busy earning income to attend. It was generally agreed that women had easier access to human health information while men could more easily learn about animal health. Training CHWs was considered highly impactful – they are trusted community members and already have a platform to disseminate knowledge widely.

*“Women are more informed... because women participate more. Men go to look for money.”* FGD, Farmers, MP

*“Everyone can access the radio, but depending on their financial ability, men generally have more money than women. This means men can access more information than women”* KII, WASH officer, MP

#### Subtheme 2: Knowledge gaps.

Participants felt that community messaging focused on human health and hygiene. Programs to educate farmers on specific zoonoses or livestock species were scarce. The few training courses on pig production targeted large-scale producers, focused on high tech services such as artificial insemination, and excluded small-holder farmers who could not afford to obtain such information directly from veterinarians. Moreover, such training courses often lacked foundational information on pig husbandry, contributing to the myth that pigs could thrive on any available foodstuff, including human waste. Participants across all nodes of the pork value chain reported serious and wide-reaching misperceptions and gaps in *T. solium* knowledge. For example, participants consistently maintained that taeniasis was transmitted by eating with unclean hands, handling pig feces and water source contamination. Information provided to a husband was not reliably disseminated to wives, perpetuating preventable production losses by keeping women uninformed.

*“Trainings are few. Trainings are provided to farmers who raise big number of pigs... farmers raising one, two or three pigs do not have trainings.”* KII, Slaughterhouse owner, MP

*“There is no special professional training for Taenia or cysticercosis.”* KII, Veterinarian, MP

*“Some people who have conflicts with their neighbors, claim to have been poisoned or say that they were bewitched, yet there are worms inside him.”* FGD, Vendors, MP

#### Subtheme 3: *Taenia solium* burden.

Livestock diseases presented a concern for smallholder farmers. *Taenia solium,* in particular, caused economic losses due to decreased market value of infected pigs, confiscated animals, and monetary fines. Such losses were devastating for smallholder producers and traders.

*“When a person’s pig is infected with* T. solium *or erysipelas, it loses value. You can’t buy it or take it for breeding… When you buy it, you must hide it, and you sell it still in hiding. If it was 100,000 RWF, you give it away at 50,000 RWF or even 30,000 RWF”.* KII, Veterinarian, MP

Participants also worried about *T. solium* infection in people, believing incorrectly that such individuals were physically weakened, struggling to work on their farms or find employment. This economic hardship was passed on to families and considered to be multi-generational. Some felt that women were already weaker than men, and therefore more impacted when infected.

*“It causes poverty for the family because someone spends about four months sleeping, doing nothing.”* FGD, Consumers, MP

*“Even when you suffer from Taenia, you will not give birth to a healthy child. The child could also be born with the disease.”* FGD, Consumers, MP

*“Women’s and men’s strengths are not equal, and the work we are doing is hard; that weakness damages your stomach and back, so that’s how it makes you weak.”* FGD, Vendors, FP

#### Subtheme 4: Community participation in *Taenia solium* prevention.

Across the pork value chain, there was a strong and recurring demand for information and resources focused specifically on pig production and *T. solium* control. Both men and women expressed interest in attending trainings and implementing solutions but identified poverty as a major barrier. In general, smallholder farmers did not follow disease prevention protocols, preferring to buy medications only when an animal appeared ill. However, farmers in this study were willing to pay for *T. solium* vaccines and anthelmintics, emphasizing the need for affordable products and accessible education.

*“If they come to teach us in our village, we will all come. We don’t want to lose pigs or health.”* FGD, Farmers, FP

*“If it is proven that it works, we can contribute…even 500 RWF [0.35 USD] each. But first, we need to understand.”* FGD, Farmers, FP

*“We need fridges and clean areas, but we are poor. I ask NGOs to support us like they support others.”* KII, Slaughterhouse owner, MP

Butchers and veterinarians noted the importance of good pig husbandry in *T. solium* control, providing both high quality foodstuffs and building appropriate infrastructure. Some smallholders agreed but could not afford improved feeds. Use of pens to confine pigs, and their cleanliness, varied across smallholder households. Such biosecurity was easier to control on larger farms with resources for veterinary care. Larger farms were also more likely to invest in improved breeds.

*“Living in dirty conditions leads to worsened health and shorter lifespans for the domestic animal.”* FGD, Consumers, MP

*“Feeding pigs the food of best quality … it cannot be infected. A pig well-fed produces meat with good taste and good appearance like meat of exotic pig not like a local pig. I have never seen infected exotic pigs, it’s rare to be infected with Taenia solium*. *Taenia solium is found in local pigs.”* KII, Slaughterhouse owner, MP

*“Encourage people to breed in shelters /farms, it’s better than the pigs meandering”* FGD, Butchers, FP

Radio and gender-specific messaging were considered impactful solutions for advertising campaigns and reaching smallholder pig producers. Moreover, there was a recognition that education campaigns targeting *T. solium* control should be multi-sectoral, providing information at different nodes that disrupted different sections of the parasite life cycle.

*“I heard on the radio about meat with white dots. That was useful. We want more such messages.”* FGD, Farmers, FP

*“We teach people that feeding pigs human waste is dangerous. We also advise pork traders not to use infected pigs and to cook meat thoroughly.”* KII, Veterinarian, MP

The interlinkages between upstream drivers, intermediate determinants and downstream outcomes in *T. solium* transmission the study region are illustrated in Fig 2.

**Fig 2:**
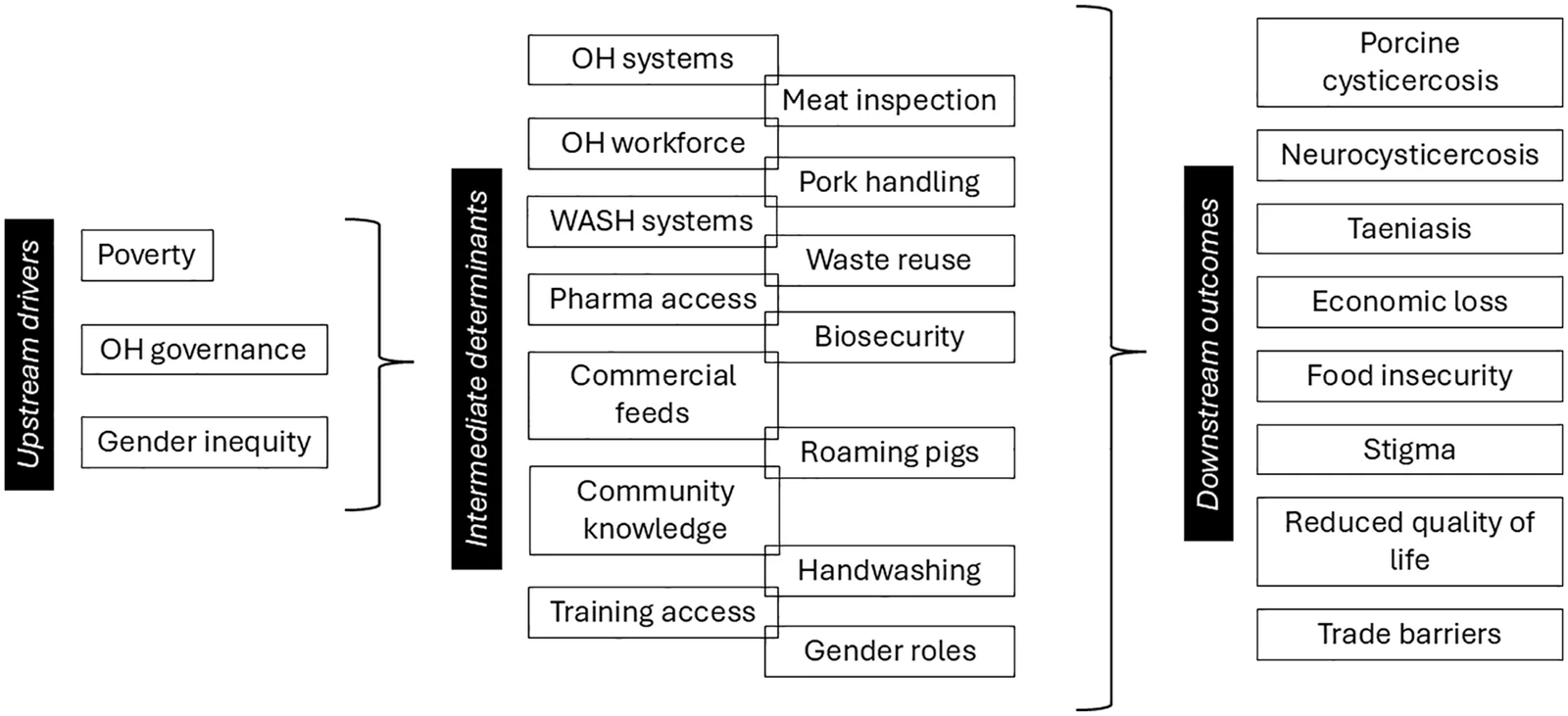
Concept map of *Taenia solium* drivers, determinants and outcomes in Nyamagabe District, Rwanda.

## Discussion

In Nyamagabe, key biosecurity vulnerabilities along the smallholder PVC included traditional production practices, poor WASH infrastructure, inadequate access to prevention services, retention of infected pigs in the food chain, circumvention of veterinary inspection at slaughter, and unhygienic consumer practices such as eating infected and/or undercooked pork. These findings, verified by local authorities, align with previous assessments of the Rwanda smallholder PVC [13,24,25] and correlate to the high prevalence of cysticercosis reported in pigs [5]. Such conditions are not unique to Rwanda. Across Southern and Eastern Africa, porcine cysticercosis has been associated with inadequate WASH infrastructure, free-roaming pig production systems, and informal slaughter practices [26]. Such sub-optimal husbandry practices have both individual and community impacts, limiting pig growth, smallholder profits and family access to animal source protein, as well as threatening animal welfare [27]. Moreover, the presence of cysticercosis in pigs means that people are also infected and coping with consequences of taeniasis and NCC. Optimizing pig husbandry in *T. solium* endemic regions provides opportunities to improve human health and livelihoods through the reduction of child malnutrition and the avoidance of medical expenditures and productivity losses.

Gender roles and responsibilities among Nyamagabe PVC smallholders were overwhelmingly traditional, suggesting that men and women experience differing modes and risk levels of *T. solium* exposure. Children grazed/fed pigs, cleaned pig pens and cleaned slaughter slabs (boys only) but rarely consumed pork, suggesting lower risk of taeniasis infection. Women’s care-giving roles put them in frequent contact with human waste while men had more contact with carcasses and pork. Meanwhile, men’s belief that alcohol consumption is protective against *T. solium* could lead to riskier eating habits and increased infection. The few epidemiological assessments of human *T. solium* infection in Rwanda do not definitively substantiate these observations. One study of Rwandese school children in Northern Province demonstrated higher cysticercosis sero-positivity among girls [7], while an NCC investigation of epilepsy patients in Southern Province showed no gender differences [8]. In neighboring Uganda, women have been shown to be over-represented among NCC patients [28]. Another key difference related to men and women’s control over and access to resources. Our data suggested that rural women often experienced an inequitable cost-benefit burden in pork production, fetching water and feed to care for pigs while having limited power to make decisions or control profits related to this work. This aligns with local reports highlighting rural women’s higher burden of unpaid work and inequitable participation in household decisions, control over finances, marketing of agricultural commodities, and access to veterinary services, bank loans or trainings [29,30]. In the traditional rural Rwandan context, biosecurity upgrades would require short-term labor inputs from men (to build WASH/pig infrastructure) and long-term labor inputs from women (to gather food, clean enclosures, maintain infrastructures). Altogether, this demonstrates the importance of gender-specific information campaigns highlighting how men and women can work together to reduce *T. solium* infection. It also suggests that normalizing women’s control over resources and bolstering self-autonomy could substantially improve family wellness. Under the COHERS program, field teams explicitly invited female participation during recruitment campaigns so that husbands would permit wives to attend.

Our study participants identified poverty and low knowledge as key limitations in implementing *T. solium* control. According to the World Health Organization, One Health efforts to reduce burden could include upgrades to latrine infrastructure, mass distribution of taenicidal medications to people (praziquantel, niclosamide) and pigs (oxfendazole), and vaccination of pigs [31]. However, impoverished and food insecure households would struggle to finance infrastructure upgrades (WASH, livestock enclosures), purchase human or veterinary medications, or adhere to veterinary regulations requiring the destruction of diseased animal carcasses or enclosure of pigs. Moreover, lack of information on the risks and benefits of such practices and concerns about stigma could further deter participation in activities intended to improve both human and animal health, even when appropriate legislation exists. The continued practice of free grazing has a clear benefit of free forage which might outweigh the risk of porcine cysticercosis in a region where slaughter is poorly regulated and zoonotic transmission risks are less known. In neighboring Uganda, oxfendazole drenching and TSOL18 vaccination have been demonstrated effective in reducing porcine cysticercosis [32,33] and were accepted by farmers when pig treatment translated to higher carcass value [34]. However, these veterinary products are not approved or available for use in Rwanda. Praziquantel to treat human taeniasis is available and regularly distributed to school aged children in *Schistosoma* endemic sectors, but this does not reliably overlap with *Taenia* endemicity. As such, adults and those residing outside *Schistosoma-*afflicted sectors currently miss out on free taenicidal prevention programming. Addressing these gaps in veterinary and public health programming could allow the government ministries to further national goals for cysticercosis control, improved pork production and poverty alleviation [17–19].

The Government of Rwanda aims for 100% access to safe water and improved sanitation by 2050 [19]. In this study, participants identified significant gaps to WASH infrastructure in rural households, marketplaces, slaughterhouses and eating establishments, directly threatening pork safety. Inadequate human and animal access to WASH has previously been documented in Nyamagabe and elsewhere, by both national census data and independent research [15,16,35]. The water scarcity, long distances, and unpredictable wait times place an especially high burden on women and children, who are predominantly responsible for fetching household water in rural Rwanda [36]. These conditions are barriers for maintaining good hygiene, preventing infections, and following safe food preparation practices. The use of raw human waste to feed pigs or fertilize food crops also has serious public health consequences, implicated in the transmission of *T. solium* and other zoonotic diseases [37]. Changing WASH infrastructure and practices is challenging for both individuals and systems. In Zambia, latrine use was closely associated with cultural taboos and social expectations, as well as practical considerations such as distance of facilities from farming areas [38]. In Rwanda, the WASH sector receives less than one-quarter of required funds to achieve their goals and is vulnerable to climate disasters [36]. Altogether, this amplifies the need for integrated multidisciplinary approaches that consider environmental infrastructure solutions in the context of human behaviour, livestock production goals, and the changing climate.

According to study participants, opportunities to improve knowledge of pig production and livestock health were scarce and some groups (women and smallholder producers) were systematically excluded. This could explain why so many misperceptions on good pig husbandry and *Taenia* infection existed. The COHERS consortium has attempted to address inequities in knowledge dissemination by hosting female voices on rural radio, facilitating gender transformative participatory theatre, and by mass dissemination of a children’s comic book describing *T. solium* prevention. Elsewhere in Africa, successful *T. solium* control has relied on inclusive One Health programming where information and resources were shared across communities, regardless of gender, farm size or wealth [39]. Key messages have focused on safe food preparation (clean utensils, washing vegetables, cooking meat), use of improved latrines, formalized pig production (confining pigs, preventing ingestion of human waste) and veterinary meat inspection, adapted to local needs and contexts [14]. This study documented both gendered *T. solium* exposure as well as gendered access to information, suggesting that education campaigns should target intended beneficiaries, providing specific information relevant to learner interests, and using suitable communication platforms. Explicitly inviting women to such events in flyers and promotions can over-ride husbands’ objections to their participation. Moreover, recommendations to change current smallholder pork production practices should consider resulting time demands for men versus women. For example, confining pigs and improving diets has benefits for disease control but creates more work for the household members tasked to care for pigs, and can also increase initial cost outputs [40]. Messaging on gender transformative approaches - recognizing the value of equitable access to resources, joint household decision-making, and the shared responsibility of infection control - provides an avenue for addressing some of the social vulnerabilities in the PVC and aligns with the Government of Rwanda’s national gender strategy [41].

This qualitative evaluation of gender roles and WASH access along the rural smallholder PVC has several limitations. First, the small number of respondents localized to one district, and nominated by local leaders, means that our conclusions should not be extrapolated to the rest of Rwanda where wealth, gender norms, pig husbandry and WASH access vary. Existing epidemiological data suggests that suitable conditions for *T. solium* transmission exist nationwide but are most common in southern and western Rwanda [5]. Although less visible individuals might have been underrepresented in this study, the diversity of demographic characteristics and roles across the PVC enabled us to capture different perspectives. Second, our study tools aimed to explore the presence and absence of facilitators and barriers for continued disease transmission, rather than to evaluate their frequency. Thus, we cannot comment on how often inappropriate behaviour occurred or how many people participated. Third, our study relied on self-reporting to identify risky practices contributing to *T. solium* transmission. Some participants might have felt embarrassed or ashamed to discuss certain practices, contributing to under-reporting. Risky practices were largely confirmed by local officials acting as key informants and/or field observations; however, we were unable to verify all information, especially those related to illicit practices. Cysticercosis-related stigma and stakeholder willingness to participate in *T. solium* control programs, such as deworming and education, should be further explored.

In conclusion, this PVC assessment highlighted key areas where the rural pork industry currently falls short in contributing to Government of Rwanda goals for poverty elimination, malnutrition reduction, NTD control, gender equity, and improved human, animal and environmental health. There is a great need for government and non-governmental groups to work across One Health disciplines to support smallholder producers in accessible training, upgrading WASH infrastructure along the PVC, expanding veterinary personnel and solutions, and investing in a community-based One Health workforce. Our results documented numerous avenues for infected pork to reach consumers, indicating an urgent need for new tools to protect the food supply chain. These could include policy changes around informal slaughter, veterinary inspection, permission to seize infected animals, and improving the availability of preventative care, such as oxfendazole and the TSOL18 vaccine.
